# Epidemiology, clinical characteristics and potential mechanism of ibrutinib-induced ventricular arrhythmias

**DOI:** 10.3389/fphar.2024.1513913

**Published:** 2024-11-19

**Authors:** Yilin Pan, Yanan Zhao, Hangyu Ren, Xintong Wang, Caixia Liu, Beibei Du, Kumaraswamy Nanthakumar, Ping Yang

**Affiliations:** ^1^ Department of Cardiology, China-Japan Union Hospital of Jilin University, Jilin Provincial Cardiovascular Research Institute, Changchun, China; ^2^ Department of Critical Care Medicine, Beijing Anzhen Hospital, Capital Medical University, Beijing, China; ^3^ Norman Bethune Health Science Center of Jilin University, Changchun, China; ^4^ The Second Affiliated Hospital of Xi’an Jiaotong University, Xi’an, China; ^5^ National Key Discipline in Hematology of China, Department of Hematology, The First Hospital of Jilin University, Changchun, China; ^6^ The Hull Family Cardiac Fibrillation Management Laboratory, Toronto General Hospital, Toronto, ON, Canada

**Keywords:** ibrutinib, ventricular arrythmias, atrial fibrillation, Bruton’s tyrosine kinase inhibitor, cardio-oncology

## Abstract

The Bruton’s Tyrosine Kinase Inhibitor, ibrutinib, has been widely employed due to its significant efficacy in B-cell lymphoma. However, the subsequent cardiac complications, notably atrial fibrillation (AF) and ventricular arrhythmias (VAs),associated with ibrutinib treatment have emerged as a major concern in cardio-oncology and hematology. Ibrutinib-induced AF has been well described, whereas mechanisms of ibrutinib-induced VAs are still under-investigation. The incidence of ibrutinib-induced VAs can vary vastly due to under-recognition and limitations of the retrospective studies. Recent investigations, including our previous work, have proposed several potential mechanisms contributing to this adverse event, necessitating further validation. The development of effective strategies for the prevention and treatment of ibrutinib-induced VAs still requires in-depth exploration. This review aims to establish a comprehensive framework encompassing the epidemiology, mechanistic insights, and clinical considerations related to ibrutinib-induced VAs. This article outlines potential strategies for the clinical management of patients undergoing ibrutinib therapy based on suggested mechanisms.

## 1 Introduction

Chronic activation of the B-cell receptor signaling pathway and its downstream overexpression as well as activation of Bruton’s Tyrosine Kinase (BTK) characterizes the pathogenesis of various B-cell non-Hodgkin’s lymphomas. Ibrutinib, the first covalent kinase inhibitor targeting BTK and downstream signaling cascades, has demonstrated remarkable clinical efficacy in extending the survival and enhancing the quality of life for affected patients (Byrd et al., 2013; Woyach et al., 2018; Wang et al., 2013; Treon et al., 2021). Due to its ability to cause significant improvement in disease prognosis, it was approved as a “breakthrough therapy” for the treatment of chronic lymphocytic leukemia (CLL), chronic myeloid leukemia, marginal zone lymphoma, and waldenstrom macroglobulinemia (Lee et al., 2016; Dreyling et al., 2016; Shanafelt et al., 2019; Treon et al., 2018). However, in addition to the typical adverse reactions neutropenia and bleeding, accumulating evidences suggest ibrutinib treatment can lead to cardiac arrhythmias (Burger et al., 2015; Lampson et al., 2017; Tuomi et al., 2018; Castillo et al., 2024; Gambril et al., 2024; Moslehi et al., 2024; Sherazi et al., 2023). This review aims to comprehensively address the epidemiology, pathogenesis, and clinical characteristics of ibrutinib-induced ventricular arrhythmias (VAs), drawing upon the latest findings in the literature. Furthermore, it seeks to present innovative strategies for clinical management founded upon an understanding of the underlying mechanisms.

## 2 Epidemiology of ibrutinib-induced VAs

Ibrutinib is the first BTK Inhibitor (BTKi) approved for clinical use and is effective against various B-cell malignancies. Currently, it is being widely used as a first-line treatment for managing CLL, chronic myeloid leukemia, marginal zone lymphoma, and waldenstrom macroglobulinemia (Brown, 2018; Aw and Brown, 2017; Burger et al., 2014; Advani et al., 2013). In several large-scale randomized controlled clinical trials, patients treated with ibrutinib exhibited significant improvements in progression-free survival, overall response rate, and overall survival compared to those in the control group (Byrd et al., 2013; Burger et al., 2015).

Nevertheless, it has been documented that ibrutinib can cause various arrhythmias, including supraventricular tachycardia, atrial fibrillation (AF), and VAs, among which AF was identified as the most prevalent manifestation. Ibrutinib-induced arrhythmias have limited its application in clinical utility (Wang et al., 2015; Herman et al., 2011; Li et al., 2023). It is essential to note that while ibrutinib-induced VAs is infrequent, they represent critical and potentially life-threatening side effects associated with ibrutinib therapy (Lampson et al., 2017; Tomcsányi et al., 2016).

### 2.1 Increased risk and incidence

Ibrutinib-induced AF was first found in the Phase III RESONATE study back in 2014 (Byrd et al., 2014). The emergence of ibrutinib-related polymorphic ventricular tachycardia (VT) and ventricular fibrillation (VF) was initially documented in a patient undergoing ibrutinib treatment in 2016 (Wallace et al., 2016). Subsequently, a spectrum of VAs, encompassing short-run VT, VF, electrical storm, and torsade de points (TdPs), have been reported in association with ibrutinib administratio n in ten documented cases (Table 1) (Tomcsányi et al., 2016; Beyer et al., 2017). A series of retrospective studies of the databases on adverse reactions have been conducted for concerns related to the safety of ibrutinib (Table 2).

**TABLE 1:** Cases of ibrutinib-associated VAs published in PubMed.

Author	Age	Sex	Type of arrhythmias	Medication time (month)	Discontinued of ibrutinib	QTc interval	Medication treatments	EP therapy	Follow-up therapy
Wallace et al. (2016)	78	Male	VT	14	Yes	Not mentioned	antiarrhythmic drug procainamide and amiodarone	-	Ibrutinib was discontinued and replaced with combination rituximab and lenalidomide therapy
Tomcsányi et al. (2016)	74	Female	VT, VF	14	Not mentioned	Not prolonged (About 400 ms)	-	ICD	Not mentioned
Beyer et al. (2017)	57	Male	ES	1.5	Yes	469 ms	Magnesium supplementation, antiarrhythmic drug lidocaine and amiodarone	Cardioversion	The patient was cannulated for ECMO on hospital day 2, on hospital day 6 he was weaned from the intra-aortic balloon pump and ECMO and discharged on hospital day 43
Lampson et al. (2017)	60	Male	VT	3	Yes	Not mentioned	antiarrhythmic drug quinidine and metoprolol	ICD	Ibrutinib was resumed at discharge, he has since been maintained for 28 months with occasional non-sustained VT on device interrogation.
	55	Male	VT、VF	12	Resume after brief outage	Not mentioned	bisoprolol	ICD	Ibrutinib was resumed 50 days after the event and he has since been maintained on ibrutinib and bisoprolol for 2 years without any additional documented episodes of VT
	58	Male	VT	1	Resume after brief outage	Not mentioned	nadolol 40 mg daily		Ibrutinib was resumed 57 days after the initial event, but 25 days later, he developed frequent PVCs during a lower respiratory tract infection, and ibrutinib was permanently discontinued
	85	Male	VF	23	Resume after brief outage	Not mentioned	bisoprolol	ICD	One month later, he was restarted on ibrutinib at 140 mg daily without subsequent cardiac events. Ibrutinib was discontinued 7 months later, after disease progression
Bernardeschi et al. (2019)	58	Male	VF	9	Yes	454 ms	Not mentioned	Cardioversion	During cardiac arrest irreversible brain damage had occurred and 4 day later, the patient was declared dead. No autopsy investigation was performed
Madgula et al. (2020)	68	Male	VT	24	Yes	About 370 ms	Antiarrhythmic drug amiodarone and metoprolol	ICD and cardioversion	On ICD interrogation at 3 months after cessation of ibrutinib, there were no further arrhythmias
Rivera et al. (2021)	67	Male	VT、AF	4	No	Not mentioned	Antiarrhythmic drug amiodarone and metoprolol	Cardioversion and subcutaneous insertable loop recorder	Ibrutinib was increased to its total dose of 420 mg daily, and the patient was started on metoprolol 12.5 mg daily and aspirin 81 mg daily. At this time, the cardiology team implanted a subcutaneous insertable loop recorder (Reveal)

VT (Ventricular Tachycardia), VF (Ventricular Fibrillation), ES (Electrical Storm), ECMO (Extracorporeal Membrane Oxygenation), ICD (Implantable Cardioverter Defibrillator), AF (Atrial Fibrillation).

**TABLE 2:** Ibrutinib-associated VAs in previous trials.

Author [ref]	Number of VAs or sudden cardiac death	Sex	Age at onset, yrs	Drug combination	Ibrutinib dose, mg/day	Time to VAs onset, days	Outcome
Chanan-Khan et al. (2016)	7	Not mentioned	Not mentioned	Ibrutinib + bendamustine + rituximab:7/7 (100%)	420:7/7 (100%)	Not mentioned	1 dead for Ventricular flutter, 2 unknown cause of death, 1 cardiac arrest leads to death, 1 sudden death
Lampson et al. (2017)	13	Not mentioned	Median: 61 Min-max: 49–85	Not mentioned	Not mentioned	Median: 65 Min-max: 6–698	Not mentioned
Guha et al. (2018)	11	Not mentioned	Not mentioned	Not mentioned	Not mentioned	Median, months: 16 Min-max, months: 0.7–57.6	Not mentioned
Cheng et al. (2018)	33	Not mentioned	Not mentioned	Only Ibrutinib: 30/33 (90.1) Ibrutinib + ≥1 other drugs: 3/33 (9.9)	≥420: 26/33 (78.8) ≤280 or not mentioned: 7/33 (21.2)	Median: 115 Min-max: 9–791	5 deaths,11 life-threatening and 21 hospitalizations events Improvement after ibrutinib reinitiation: 19 cases
Salem et al. (2019)	70	Male:49/67 (73.1) Female:18/67 (26.9)	Mean ± SD: 65.3 ± 12.4 Min-max: 8–85	Only Ibrutinib: 57/70 (81.4) Ibrutinib +1 other drug: 7/70 (10.0) Ibrutinib + ≥2 other drugs: 6/70 (8.6)	140: 2/56 (3.6) 280: 3/56 (5.4) 420: 40/56 (71.4) 560: 10/56 (17.8) >560: 1/56 (1.8)	Median: 70 Min-max: 1–1,002	Death: 7/70 (10.0)
Moreno et al. (2019)	6	Not mentioned	Not mentioned	Ibrutinib + Obinutuzumab:6/6 (100%)	420:6/6 (100%)	Not mentioned	1 sudden death, 1 cardiac arrest leads to death., 1 death due to cardiac arrest after cross over to ibrutinib arm, 3 unknown cause of death
Brown et al. (2023)	8	Not mentioned	Not mentioned	Only Inrutinib:8/8 (100%)	420:8/8 (100%)	Not mentioned	2 cardiac arrest lead to death

VAs (Ventricular Arrhythmias), SD (Standard Deviation).

Lampson et al. (2017) conducted an analysis of the Food and Drug Administration (FDA) Adverse Event Reporting System (FAERS) data ranging from the 4^th^ quarter of 2013 to the 4^th^ quarter of 2015, they identified 13 patients who developed various VAs after ibrutinib treatment. The calculated weighted incidence rate of ibrutinib-induced VAs was 788 people/100,000 person-years, significantly higher (2.0 to 3.9-fold) than the age-matched incidence in the general population (200–400/100,000 person-years) (Lampson et al., 2017; Becker et al., 1993; Chugh et al., 2004; Rothwell et al., 2005). Additionally, in a retrospective study by Guha et al. (2018), 582 patients who were administered ibrutinib, 11 patients developed symptomatic VAs presumably related to ibrutinib therapy. Notably, six patients without pre-existing cardiac disease developed VAs, resulting in a calculated weighted incidence of 596 per 100,000 person-years, which was significantly higher (expected Relative Risk 12.4) than the average population (48.1 per 100,000 person-years) (Sirichand et al., 2017). In 2018, an updated query of FAERS (Data from November 2013- November 2017) by Cheng et al. (2018) identified 33 cases of VAs (based on the WHO/UMC causality assessment scale) possibly associated with ibrutinib treatment. The causal relationship between ibrutinib and VAs was determined as probable in 8 patients without established confounding factors, such as electrolyte imbalance or other QT-prolongation medication. Although the rest of the 25 patients were identified as possible casualties, it is noteworthy that a positive dechallenge concerning ibrutinib discontinuation or positive rechallenge regarding ibrutinib reinitiating indicated that ibrutinib was directly associated with VAs. An extensive analysis of the Vigibase, the international pharmacovigilance database conducted by Salem et al. (2019), delved into the cardiotoxic effects of ibrutinib. This investigation identified 70 patients, comprising 31 cases of VT and 20 instances of VF, where ibrutinib was linked to the induction of VAs. Ibrutinib was found to be closely associated with a calculated higher incidence of VAs (reported odds ratio [ROR]: 4.7; 95% CI: 3.7–5.9; *p* < 0.0001). A recent study led by Zheng et al. (2023) conducted a comprehensive analysis of the FAERS database covering 2014 to 2021. This investigation shed light on the frequent occurrence of cardiotoxic events, specifically VT and VF, associated with using ibrutinib. The analysis yielded a substantial information component value of 1.18 and a noteworthy ROR of 2.27 for VT. Within the spectrum of cardiovascular toxicities, torsade de pointes/QT prolongation (TdP/QTp) represented the category with the highest fatalities. The information component and ROR values for TdP/QTp were 0.17 and 1.12, respectively.

Early evidence from the literature provides us with a preliminary estimation of the incidence of this critical arrhythmia. Insights gleaned from prescription data of ibrutinib and results from a small randomized controlled trial with IMBRUVICA provide informative. In this trial, spanning a median follow-up of 19.5 months, the incidence of VAs, including ventricular premature beats, VT, ventricular flutter, and VF, among all Common Terminology Criteria for Adverse Events (CTCAE) grades, was 1.0% in the ibrutinib group (n = 1,157) and 0.4% in the control group (n = 958). The incidence rates for CTCAE grade 3 and above were 0.3% and 0%, respectively (IMBRUVICA, 2022). In a phase Ⅲ clinical trial conducted by Woyach et al. (2018), unexpected mortality was observed in 1.1% of patients in the chemoimmunotherapy group and 3% of those treated with ibrutinib. This discrepancy in mortality rates could potentially be attributed to life-threatening VF.

Interestingly, in contrast to the relatively low reported incidence ranging from 0.3% to 3%, a recent retrospective study employing ambulatory electrocardiogram (ECG) monitoring during ibrutinib treatment revealed a notably higher incidence of VAs among treated patients (Fazal et al., 2021). This retrospective study found that 43% of the 72 patients monitored had experienced non-sustained VT during ibrutinib treatment, with most cases asymptomatic. Notably, the study also highlighted the possibility of significant underestimation of asymptomatic non-sustained VT occurrences due to the absence of continuous monitoring. However, it is essential to acknowledge that this study had limitations, including a lack of information regarding patients’ arrhythmia history before ibrutinib treatment. The presence of an extraordinary higher pre-existing AF history (incidence 13%) might have contributed to an overestimation of VAs in this small-scale study.

These studies have consistently reaffirmed a significant increase in the risk of VAs following exposure to ibrutinib. However, it is crucial to note that these studies did not provide a comprehensive depiction of the incidences of ibrutinib-induced VAs, as they did not encompass the entire population exposed to the drug. Due to the under-recognition of this side effect and limitations inherent in reporting systems, the perceived risk was initially assumed to be relatively low. Subsequently, with the establishment of cohorts based on real-world data and clinical trial findings, Health Canada took action by posting information on 26 July 2018, regarding the results of an assessment of the risk of severe and life-threatening arrhythmias, particularly ventricular tachycardia, induced by ibrutinib. This information was disseminated to medical professionals and patients via InfoWatch (Canada, 2018). In May 2022, the FDA revised the instructions and guidance for utilizing ibrutinib. Notably, the updated guidance explicitly underscores the occurrence of severe and fatal cardiac arrhythmias as a prominent and significant adverse outcome associated with this medication. This heightened emphasis reflects the growing awareness within the medical community of the cardiac safety concerns linked to ibrutinib use (Canada, 2018).

### 2.2 Age, pre-existing cardiac disease, and time to event

Lampson’s study examined 13 cases of ibrutinib-induced VAs, reporting a median time from the start of ibrutinib treatment to the occurrence of VAs as 65 days, with a range spanning from 6 to 698 days (Lampson et al., 2017). The median age of patients in this study was 61 years, ranging from 49 to 85 years. Importantly, comprehensive cardiac evaluations revealed no pre-existing cardiac causes for VAs in these cases.

Similarly, Cheng et al. (2018) conducted a study involving 33 patients, identifying a median onset time of 115 days for VAs, with a range from 9 to 791 days. Notably, nine patients developed VAs within the first month of initiating ibrutinib therapy. Coronary angiography and cardiac function assessments for these patients showed no severe ischemic heart disease, and eight of these patients maintained normal left ventricular ejection fractions.

In addition, Guha et al. (2018) reported a median time to VA onset of 16 months, with a range from 0.7 to 57.6 months, in their study. Over a 32-month follow-up period, symptomatic VAs were typically observed within 11 months. Notably, seven patients showed a presumed association with ibrutinib, and six of these patients had no prior history of coronary artery disease or heart failure with reduced ejection fraction.

Synthesizing these findings, it appears that ibrutinib-induced VAs are more likely to occur in older patients, particularly those over 60. However, the timing of VA onset varies considerably, ranging from early occurrence (within 65 days) to much later detection (up to 16 months in long-term follow-up). Additionally, a significant portion of patients experiencing VAs had no prior cardiovascular disease (CVD), emphasizing the multifactorial and complex nature of these events. Despite these findings, the potential influence of factors such as region, race, or other demographic variables on VA risk remains unclear due to the limited diversity in the current body of research.

### 2.3 Dose

In C57BL/6 mice, it has been demonstrated that a single high dose of ibrutinib is more prone to induce arrhythmias than a low dose. However, no dose association has been observed in cases of chronic administration. Instead, the incidence of arrhythmias may be more related to plasma concentration (Tuomi et al., 2018). Moreover, in clinical data, no significant correlation has been identified between the incidence of arrhythmias and the administered dose of ibrutinib.

Cheng et al. (2018) reported the contributory role of ibrutinib dose on VAs in 33 patients. They observed that whether patients were treated with the FDA-recommended doses of 420 mg/day, 560 mg/day, or even a reduced dose of 280 mg/day, there was no clear correlation between the dose administered and the occurrence of VAs. This assessment considered hepatic metabolism parameters, and no evidence of hepatic dysfunction or cytochrome P450 3A inhibition was found, as indicated in the primary drug safety evaluation report (IMBRUVICA, 2022). Similarly, an analysis of VigiBase data by Salem et al. (2021) revealed that the administration of 420 mg/day or 560 mg/day doses accounted for a significant portion of total ibrutinib-induced VAs, with 71.4% and 17.8%, respectively. This observation further suggests a loose association between different doses of ibrutinib and the occurrence of ibrutinib-induced VAs. These findings underscore the complex nature of ibrutinib-associated arrhythmias, where factors other than the specific dose may exert a more substantial influence.

### 2.4 Comparison with ibrutinib-induced AF

**FIGURE 1:**
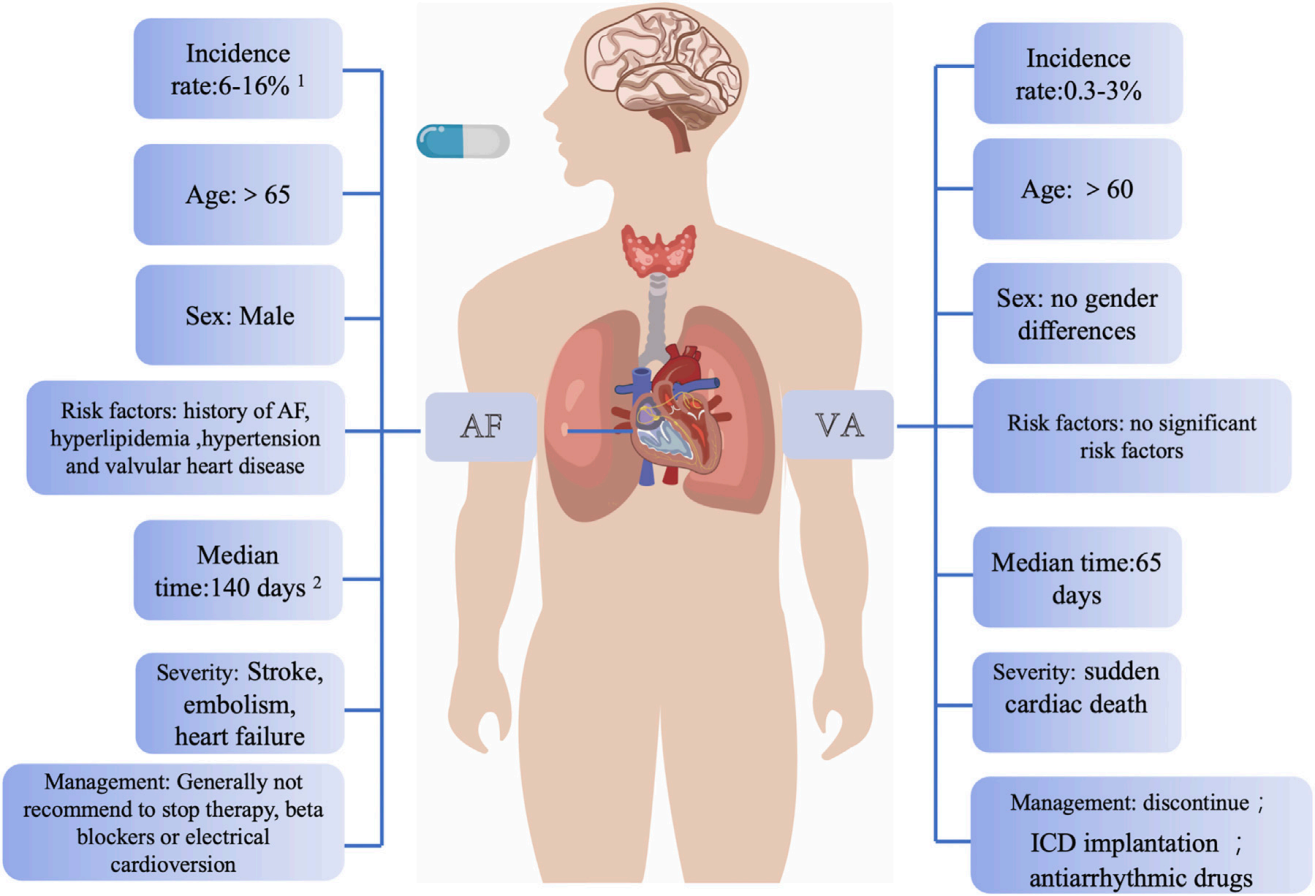
Main clinical differences between ibrutinib-associated AF and VAs. This figure highlights that AF typically occurs in older patients with extended ibrutinib use and is associated with male gender and a history of AF, whereas VAs are more acute, life-threatening, and often arise in patients without pre-existing cardiovascular disease. VAs generally necessitate discontinuing ibrutinib, while AF may allow dose adjustments. Data sources: ^1.^ Characterization of AF events in ibrutinib trials; ^2.^ Long-term follow-up of MCL patients on single-agent ibrutinib. Abbreviations: AF, atrial fibrillation; VAs, ventricular arrhythmias; ICD, implantable cardioverter defibrillator.

Ibrutinib was approved for treating relapsed/refractory CLL by the FDA in 2014 (Mato et al., 2022). Extensive research has explored the association between ibrutinib and AF. It has been firmly established that AF is the most prevalent cardiac complication associated with ibrutinib (Christensen et al., 2022; Buske et al., 2023). In contrast, VAs represent are rare but represent one of the most severe types of arrhythmias induced by ibrutinib. These two cardiac complications can be differentiated from each other based on numerous similarities, including age, incidence, and their relationship to the primary disease (Figure 1).

In various epidemiological studies on AF caused by ibrutinib, advanced age (>65 years) was identified as a risk factor for AF after medication, and the incidence of AF was positively correlated with the duration of medication (Wang et al., 2013; Burger et al., 2014; Tang et al., 2018; Brown et al., 2017; O'Brien et al., 2014; Byrd et al., 2015; Shanafelt et al., 2017). The available evidence from various case reports and retrospective studies regarding VAs induced by ibrutinib therapy does not provide conclusive support for a significant difference in the age of affected patients. Some retrospective studies have reported a median age of over 60 for individuals experiencing ibrutinib-associated VAs. Our previous basic research also suggested a higher occurrence of VAs in older individuals (Du et al., 2020). However, it is crucial to consider the potential inclusion bias associated with the age at the onset of CLL, which typically occurs in individuals with a median age ranging between 67 and 72 (Yang et al., 2021; Strati and Shanafelt, 2015). This inherent age factor in CLL patients may influence the observed age distribution in ibrutinib-related VAs cases. In summary, while existing evidence suggests a possible increased risk of VAs in older individuals, drawing definitive conclusions regarding age-related differences requires further investigation through large-scale clinical studies that carefully account for CLL onset age and other potential confounding factors.

Various studies have pinpointed several potential risk factors for developing AF, including age, male, a prior history of AF, hyperlipidemia, hypertension, and valvular heart disease (Brown et al., 2017; Shanafelt et al., 2017; Wiczer et al., 2017; Thompson et al., 2016). In the context of ibrutinib-induced VAs, limited evidence suggested that male gender, wide QRS, a history of AF, diabetes, heart failure, coronary artery disease, and valvular heart disease could be associated with these arrhythmias (Guha et al., 2018). It is noteworthy that a substantial body of evidence contradicts these findings. A significant proportion of patients who develop VAs after ibrutinib treatment have been reported to have no pre-existing CVD or traditional cardiovascular risk factors.

AF can lead to impaired functional capacity and increase the risk of stroke. However, it is essential to emphasize that this clinical challenge can often be effectively managed and even resolved through a collaborative and multidisciplinary approach involving hematologists and cardiologists. International retrospective analyses have also suggested that patients who experience the onset of AF while on ibrutinib treatment and have their ibrutinib dose either reduced without interruption or continue with the total amount may achieve better progression-free survival compared to those who have their ibrutinib treatment interrupted (Thompson et al., 2016). In contrast, the symptoms associated with VAs can be more acute, carry a higher risk, and are potentially life-threatening compared to AF (Fazal et al., 2023; Wang and Guo, 2022). Due to the substantial mortality rate associated with VAs, it is recommended that patients experiencing VAs should immediately discontinue ibrutinib therapy (Tang et al., 2022). This highlights the critical need for vigilant monitoring and rapid intervention in cases of VAs to ensure patient safety.

## 3 Clinical characteristics of ibrutinib-induced VAs

### 3.1 Symptoms and severity

The symptoms of ibrutinib-induced VAs can vary from palpitation, dizziness, lightheadedness, and chest discomfort to loss of consciousness and sudden cardiac death (SCD) (Tang et al., 2022). A high SCD rate has been reported in several prior cases and studies. Lampson et al. (2017) study identified that the SCD incidence rate of ibrutinib was 788 events per 100,000 person-years. In the HELIOS study, this rate reached 1991 events per 100,000 person-years. One of the key challenges in assessing VAs is the absence of clear and standardized definitions and indicators (Fazal et al., 2023; Chanan-Khan et al., 2016). Cardiac monitors can detect non-persistent ventricular tachycardia, irregular ventricular premature beats, and polymorphic ventricular tachycardia such as Tdp, which can evolve into VF in severe cases (Fazal et al., 2021; Fazal et al., 2023). Once these arrhythmias occur, they often arise continuously during ibrutinib treatment and induce fatal adverse prognosis. Hence, early monitoring and identification of the occurrence of VAs is vital to improve the prognosis.

### 3.2 QT interval changes associated with ibrutinib exposure

Several tyrosine kinase inhibitors have been associated with QT interval prolongation, including nilotinib, dasatinib, and sunitinib (Lu et al., 2012). However, the potential of ibrutinib to prolong the QT interval, leading to VAs, has not been definitively established.

Beyer et al. (2017) reported that a patient undergoing treatment with ibrutinib was found to have a prolonged QT interval of 590 ms and subsequently experienced pleomorphic VAs and electrical storms. QT interval prolongation has also been reported in cardiovascular events in standardized MedDRA Query with disproportionality analysis in the FDA FAERS. (Zheng et al., 2023). However, In a small-size retrospective study, Fradley et al. (2020) found that after applying ibrutinib, both the QT interval and the corrected QT were significantly shortened. Indeed, several studies have suggested that ibrutinib may not significantly prolong the QT interval (Essa et al., 2022). In a study on the application of ibrutinib in healthy subjects, short-term administration of conventional and high-dose ibrutinib did not produce a clinically meaningful prolongation of the QT interval (de Jong et al., 2017). Even in patients monitored with non-persistent ventricular tachycardia and other VA events, no substantial effect of ibrutinib on QT interval has been detected (Lampson et al., 2017; Fazal et al., 2021).

Overall, several factors, such as enrolled patient cohort and drug combination, can significantly affect the change of QT interval. The prevailing consensus among studies is that ibrutinib therapy is not typically associated with QT interval prolongation (Salem et al., 2021).

### 3.3 Management of ibrutinib-induced VAs

During clinical treatment, patients who experience symptoms such as palpitations, dizziness, or syncope associated with VAs during ibrutinib therapy are commonly recommended to undergo ambulatory ECG monitoring and implantable ECG monitoring as diagnostic tools. (Guha et al., 2018; Bhat et al., 2022; Solbiati et al., 2017; Merner et al., 2008; Sen-Chowdhry et al., 2007). Rivera et al. (2021) reported that a patient undergoing ibrutinib treatment for CLL developed hemodynamically unstable tachyarrhythmias after 4 months of therapy. Following the implantation loop recorder and an adjustment to the treatment regimen, the arrhythmia episodes were effectively terminated, and the CLL achieved complete remission. This underscores the critical role of advanced monitoring techniques and tailored treatment adjustments in managing ibrutinib-induced arrhythmias and optimizing patient outcomes.

Ibrutinib is mainly metabolized by cytochrome P450 3A4 (CYP3A4) enzymes (IMBRUVICA, 2022). When combined with CYP3A4 inducers or inhibitor, significant inter-drug interactions may occur, potentially altering drug toxicity and efficacy (de Jong et al., 2015; Scheers et al., 2015). Combining ibrutinib with duvelisib, a moderate CYP3A4 inhibitor, can increase the concertation of ibrutinib, leading to potential unexpected adverse outcomes, including fatalities (Paul et al., 2023). As a precautionary measure, it is strongly recommended to avoid the concurrent use of ibrutinib with drugs that interact with CYP3A4. If such combinations are deemed necessary, the ibrutinib dose should be reduced, and its blood concentrations closely monitored.

VAs is often characterized by insidious onset, rapid progression, and high mortality. Arrhythmias, including VAs, constitute one of the primary reasons for discontinuing ibrutinib therapy. In a study that investigated various types of arrhythmias induced by ibrutinib, it was discovered that among the patients who experienced VAs, 25% had to discontinue ibrutinib due to arrhythmias (Tuomi et al., 2018). By current guidelines, patients who develop signs and symptoms of VAs while on ibrutinib therapy are strongly advised to discontinue the drug. Following discontinuation, a comprehensive clinical risk/benefit assessment should be conducted before potentially considering the reinitiation of the same therapy. Clinicians should maintain a high degree of vigilance when patients on ibrutinib exhibit suspected symptoms of VAs, such as dizziness, palpitations, syncope (Koplan and Stevenson, 2009). Furthermore, for patients with a confirmed diagnosis of VAs, efforts should be made to identify and address any underlying predisposing factors for VAs. In cases of VAs without hemodynamic disturbance, treatment options may include administering medications such as lidocaine, beta-blockers, or intravenous amiodarone as a bolus injection. It is important to note that antiarrhythmic drugs can potentially cause or exacerbate arrhythmias, so close monitoring of vital signs is essential during medication administration (Jamshidi et al., 2012; Kudenchuk et al., 2017; Rajan et al., 2017; Zeppenfeld et al., 2022). For patients with VAs accompanied by hemodynamic disturbances, rapid electrical cardioversion should be implemented when necessary, following administration of drugs like Amiodarone or lidocaine, which can prevent the recurrence of VAs in a short time. Implantable cardioverter defibrillator implantation therapy can be an option for patients diagnosed with VAs. In cases of recurrent VAs or repeated shocks following implantable cardioverter defibrillator implantation, catheter ablation therapy should be considered (Al-Khatib et al., 2017). These strategies are customized to the severity context of each patient’s condition, presenting a valuable contribution to the management and treatment of VAs induced by ibrutinib.

New generation BTKi, including acalabrutinib and zanubrutinib, have shown promising outcomes in CLL treatment. Preliminary cohort studies have indicated a lower incidence of AF associated with these newer BTKi options (Byrd et al., 2021; Hillmen et al., 2020). Consequently, acalabrutinib has been considered a viable alternative for patients who need to discontinue treatment with ibrutinib due to adverse events (Ferrajoli et al., 2021; Furman et al., 2021). Although the newer generation of BTKi is generally believed to have a reduced occurrence of cardiovascular toxicities such as AF and VT, recent studies has demonstrated that acalabrutinib can also induce VT and SCD (Bhat et al., 2022; Brown et al., 2022; Zhai et al., 2023). Our recent publication also indicated increased ventricular vulnerability in *in vivo* SD rat model treated with acalabrutinib (Zhao et al., 2024a). Pirtobrutinib, a noncovalent BTKi, has shown impression efficacy in relapsed CLL and fewer adverse cardiovascular events (De, 2023; Gomez et al., 2023; Mato et al., 2021). However, due to the limited time since their introduction to the market and insufficient data from clinical trials, ongoing investigations are needed to determine whether the newer generation BTKi effectively reduces the incidence of VAs. Comprehensive research and extensive clinical studies are essential to establish the safety profile of these agents concerning cardiovascular toxicities.

Awan et al. (2022) convened an International Steering Committee of 12 physicians to design practical recommendations for optimal treatment strategies in patients receiving BTKi and effective ways to mitigate cardiovascular toxicity. They recommended that the caregivers thoroughly assess a patient’s level of cardiovascular risk before initiating treatment. This assessment should encompass not only established CVD but also potential risk factors. It should be tailored based on the patient’s preexisting conditions, risk factors, a baseline ECG and a detailed cardiac history. For those with a significant cardiac history or risk factors, echocardiography is also recommended as part of the baseline evaluation. Patients should be informed beforehand about the potential risk of rare but fatal VAs and should be vigilant for symptoms. Regular monitoring, including ECG and rhythm monitoring, should be conducted to screen for VAs (Tang et al., 2022; Quartermaine et al., 2023; Paydas, 2019). Some researchers have proposed that patients who develop VAs or SCD while on therapy should not be re-challenged, as there are prior case reports of recurrent ventricular events in patients in whom the medication was reintroduced (Boriani et al., 2023).

Overall, the symptoms of VAs can vary. In instances where patients are asymptomatic or experience only palpitations, increased vigilance becomes imperative. Holter and implantable cardiac monitors can significantly enhance monitoring capabilities in such cases, aiding in the detection of subtle or intermittent arrhythmias. For VAs leading to hemodynamic compromise or induce syncope, discontinuing ibrutinib is crucial. Whether the newer BTKi can serve as an effective alternative requires further research and evaluation. Close attention should be paid to potential interactions and appropriate monitoring when using BTKi with drugs metabolized by CYP3A4. The occurrence of VAs is a complex and severe clinical issue. Thus, a multidisciplinary approach involving hematologists and cardiologists is required to facilitate optimal treatment and care for affected patients. This collaborative approach ensures the best possible management and outcomes for individuals receiving BTKi therapy.

## 4 Potential mechanism of ibrutinib-induced VAs

Structural changes in the myocardium induced by ibrutinib may constitute the basis of electrophysiology remodeling and VAs. In an *in vivo* mice study, echocardiography after 4 weeks of ibrutinib administration showed a significant increase in left ventricular (LV) diameter, indicating a trend of LV enlargement (Jiang et al., 2019). In a cohort of 33 patients treated with ibrutinib who underwent cardiac magnetic resonance imaging (MRI), 54.8% displayed late gadolinium enhancement, a marker of myocardial fibrosis. Remarkably, this subgroup of patients experienced disproportionately higher rates of subsequent cardiac events. In one typical case, a patient undergoing ibrutinib treatment exhibited myocardium inflammation and discrete fibrosis on cardiac MRI just 2 months into therapy, which was closely linked to the patient’s SCD (Buck et al., 2023).

Electrophysiological and molecular mechanisms of ibrutinib-induced VAs in preclinical models are summarized as follows.

### 4.1 Electrophysiological mechanisms

Increased autonomicity and repolarization disorders are two possible electrophysiological mechanisms. An *ex-vivo* study on cardiomyocytes found that ibrutinib induced abnormal action potentials and increased late sodium currents, leading to enhanced cardiomyocyte automaticity (Yang and Roden, 2015). In our previous study using rat models, we investigated the electrophysiological mechanisms behind VAs triggered by acute ibrutinib administration (Du et al., 2020). In the Langendorff-perfused rat model, acute exposure to ibrutinib induced more prominent action potential duration (APD) alternans and APD alternans spatial discordance, both of which are markers of membrane electrophysiological instability strongly associated with the onset of VAs. Additionally, calcium mapping in this model revealed dysregulated calcium handling, characterized by increased calcium release (shortened time-to-peak and a lower calcium amplitude alternans ratio) and impaired calcium uptake (prolonged calcium transient duration 50). Further *in vivo* study also confirmed membrane repolarization heterogeneity and calcium dynamics dysfunction (Zhao et al., 2024b). Voltage mapping revealed more prevalent epicardial discordant APD alternans, a key marker of repolarization heterogeneity. Additionally, significant alterations in calcium dynamics were observed, including prolonged decay time constants of calcium transients, reduced amplitude alternans ratios, and increased spontaneous intracellular calcium elevations. These changes in calcium handling resulted in elevated diastolic intracellular calcium levels and heightened ventricular vulnerability.

In summary, membrane repolarization heterogeneity and dysregulated calcium dynamics constitutes the electrophysiological mechanism of ibrutinib induced VAs.

### 4.2 Molecular mechanisms

With evidence of impaired calcium dynamics, the investigation of the underlying molecular mechanisms centered on calcium-handling proteins and the upstream kinases that may be regulated by ibrutinib.

The dysfunction of calcium dynamics represents a critical downstream link that cannot be ignored in the context of Ibrutinib-related arrhythmias, whether atrial or ventricular. In a study conducted by Jiang et al. where ibrutinib was administered to C57BL/6 mice for 4 weeks, increased activity of calmodulin-dependent protein kinase II and the inhibition of ryanodine receptor 2-ser2814 and phospholamban-Thr17 were identified as potential contributors to ibrutinib-induced atrial structural remodeling and calcium dysregulation (Jiang et al., 2019).

For the upstream kinase affected by ibrutinib, various study explored different pathways. Early-stage studies ruled out the phosphatidylinositol 3-kinase-protein kinase B (PI3K-Akt) signaling pathway, a critical downstream effector of B-cell receptor signaling in the treatment of blood malignancies, as the underlying mechanism for ibrutinib-related AF (McMullen et al., 2014; Pretorius et al., 2009). Notably, the PI3K inhibitor idelalisib, which explicitly targets PI3K, has not exhibited cardiotoxic side effects (Nair and Cheson, 2016; Chung and Lee, 2014). Furthermore, in another *in vivo* study on C57BL/6 mice treated with ibrutinib/acalabrutinib (Xiao et al., 2020), researchers observed an increased susceptibility to atrial fibrosis and AF in the ibrutinib group, but not in the more specific BTKi, acalabrutinib, thus reaffirming the previously reported findings that off-target effects primarily caused ibrutinib-related arrhythmias. This *in vivo* research suggested that CSK is a critical molecule that inhibits and acts as a mediator for ibrutinib-related AF.

**FIGURE 2:**
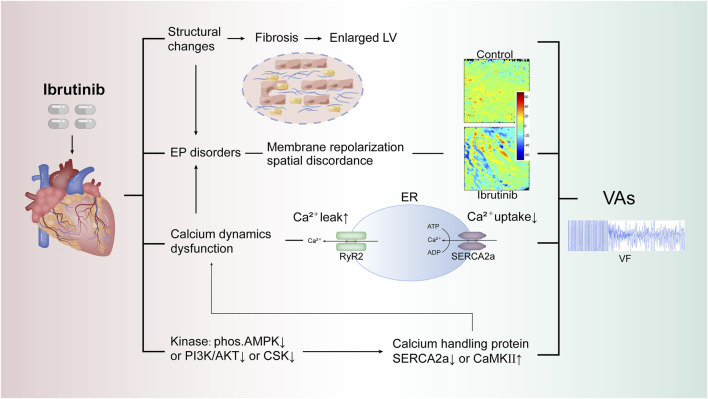
Proposed mechanisms of ibrutinib-induced VAs. This figure illustrates potential mechanisms of ibrutinib-induced VAs, focusing on key pathways involved in calcium dysregulation and electrophysiological instability. Dysregulated calcium handling, impaired SERCA2a function, and abnormal RyR2 activation are central to arrhythmogenesis. The role of upstream kinases (e.g., AMPK, PI3K/AKT) in modulating these processes and the impact of endoplasmic reticulum stress on calcium dynamics are also highlighted, increasing susceptibility to VAs. Abbreviations: VAs, ventricular arrhythmias; LV, left ventricle; EP, electrophysiological; RyR2, ryanodine receptor 2; SERCA2a, sarcoplasmic/endoplasmic reticulum calcium ATPase 2a; ER, endoplasmic reticulum; AMPK, AMP-activated protein kinase; PI3K/AKT, phosphoinositide 3-kinase/protein kinase B; CSK, C-terminal Src kinase; CaMKII, calcium/calmodulin-dependent protein kinase II; VF, ventricular fibrillation.

The energy metabolism pathway is another critical aspect to consider. The therapeutic effect of ibrutinib in lymphoma is closely associated with mitochondrial dysfunction, impacting the AMP-activated protein kinase (AMPK) signaling pathway, altered mitochondrial redox status, and cellular energy depletion (Sharif-Askari et al., 2019). Metabolic stress levels have also been linked with abnormal calcium release, diastolic calcium overload, and the influence of glycolytic intermediates on ryanodine receptor 2 calcium release. Additionally, ATP deficiency can result in decreased sarcoplasmic/endoplasmic reticulum Ca^2^⁺-ATPase 2a (SERCA2a) activity. Several previous studies have also established the role of metabolic stress levels in arrhythmias, particularly AF (Wiersma et al., 2019; Ozcan et al., 2019; Chakraborty et al., 2020). While molecular changes were not evident in the acute exposure study, our *in -vivo* findings confirmed that ibrutinib inhibits AMPK phosphorylation (Du et al., 2020; Zhao et al., 2024b). Since AMPK enhances SERCA2a expression, this inhibition significantly decreases SERCA2a levels (Morissette et al., 2019). The reduction in SERCA2a serves as a critical link between AMPK inhibition and impaired calcium handling. This dysregulation in calcium handling further contributes to membrane repolarization discordance, creating a substrate for electrical instability and increasing the risk of ibrutinib-induced VAs (Figure 2). Further validation has been provided by us using 5-Aminoimidazole-4-carboxamide ribonucleotide, an AMPK activator, which has been shown to reduce the vulnerability to ibrutinib-induced VAs (Zhao et al., 2024b). These findings highlight the potential of AMPK as a therapeutic target for managing ibrutinib-induced VAs in the future.

## 5 Conclusion

Among the cardiovascular complications arising from ibrutinib therapy, VAs are the most severe, yet not uncommon, and are associated with a poor prognosis. High-risk patients should undergo periodic screening to enable early detection of related symptoms. Timely intervention and improved clinical management are crucial for altering the prognosis. Effective collaboration between hematologists and cardiologists is essential to developing individualized treatment plans, including decisions regarding ibrutinib continuation or discontinuation, as well as arrhythmia management. Impaired AMPK activity leading to calcium handling dysfunction and ventricular repolarization discordance suggests increased vulnerability to ibrutinib-induced VAs.

## Abbreviations

AF: atrial fibrillation; AMPK: AMP-activated protein kinase; APD: action potential duration; BTK: Bruton’s Tyrosine Kinase; BTKi: Bruton’s Tyrosine Kinase Inhibitor; CLL: chronic lymphocytic leukemia; CTCAE: Common Terminology Criteria for Adverse Events; CVD: cardiovascular disease; CYP3A4: cytochrome P450 3A4; ECG: electrocardiogram; FAERS: FDA Adverse Event Reporting System; FDA: Food and Drug Administration; LV: left ventricular; MRI: magnetic resonance imaging; PI3K: phosphatidylinositol 3-kinase; PI3K-Akt: phosphatidylinositol 3-kinase-protein kinase B; ROR: reported odds ratio; SCD: sudden cardiac death; SERCA2a: sarcoplasmic/endoplasmic reticulum Ca^2^⁺-ATPase 2a; TdPs: torsade de points; VAs: ventricular arrhythmias; VF: ventricular fibrillation; VT: ventricular tachycardia.
